# Salicylic acid-induced ROS production by mitochondrial electron transport chain depends on the activity of mitochondrial hexokinases in tomato (*Solanum lycopersicum* L.)

**DOI:** 10.1007/s10265-019-01085-y

**Published:** 2019-02-13

**Authors:** Péter Poór, Gábor Patyi, Zoltán Takács, András Szekeres, Nikolett Bódi, Mária Bagyánszki, Irma Tari

**Affiliations:** 1 Department of Plant Biology, University of Szeged, Közép fasor 52, Szeged, 6726 Hungary; 2 Department of Microbiology, University of Szeged, Közép fasor 52, Szeged, 6726 Hungary; 3 Department of Physiology, Anatomy and Neuroscience, University of Szeged, Közép fasor 52, Szeged, 6726 Hungary

**Keywords:** Cytochrome *c*, Glucose, Hexokinase, Mitochondria, Salicylic acid, Tomato

## Abstract

The growth regulator, salicylic acid (SA) plays an important role in the induction of cell death in plants. Production of reactive oxygen species (ROS) by mitochondrial electron transport chain (mtETC), cytochrome *c* (cyt *c*) release from mitochondria and loss of mitochondrial integrity can be observed during cell death execution in plant tissues. The aim of this work was to study the putative role of hexokinases (HXKs) in the initiation of cell death using tomato (*Solanum lycopersicum* L.) leaves and mitochondria isolated from plants exposed to a sublethal, 0.1 mM and a cell death-inducing, 1 mM concentrations of SA. Both treatments enhanced ROS and nitric oxide (NO) production in the leaves, which contributed to a concentration-dependent loss of membrane integrity. Images prepared by transmission electron microscopy showed swelling and disorganisation of mitochondrial cristae and vacuolization of mitochondria after SA exposure. Using post-embedding immunohistochemistry, cyt *c* release from mitochondria was also detected after 1 mM SA treatment. Both SA treatments decreased the activity and transcript levels of HXKs in the leaves and the total mtHXK activity in the mitochondrial fraction. The role of mitochondrial hexokinases (mtHXKs) in ROS and NO production of isolated mitochondria was investigated by the addition of HXK substrate, glucose (Glc) and a specific HXK inhibitor, *N*-acetylglucosamine (NAG) to the mitochondrial suspension. Both SA treatments enhanced ROS production by mtETC in the presence of succinate and ADP, which was slightly inhibited by Glc and increased significantly by NAG in control and in 0.1 mM SA-treated mitochondria. These changes were not significant at 1 mM SA, which caused disorganisation of mitochondrial membranes. Thus the inhibition of mtHXK activity can contribute to the mitochondrial ROS production, but it is not involved in NO generation in SA-treated leaf mitochondria suggesting that SA can promote cell death by suppressing mtHXK transcription and activity.

## Introduction

Salicylic acid (SA), a natural phenolic compound controls many physiological and biochemical functions in plants such as growth, development or defence reactions against biotic and abiotic stressors (Hayat et al. 2010; Horváth et al. 2007; Khan and Khan 2013; Khan et al. 2015; Rivas-San Vicente and Plasencia 2011). It plays a role by time- and concentration-dependent manner in the formation of local- and systemic acquired resistance (SAR) and in the initiation of the hypersensitive response (HR) after infection with biotrophic pathogens (Yan and Dong 2014). HR is characterized by a localized cell death at or around the point of pathogen entry, which is accompanied by SA accumulation and production of reactive oxygen and nitrogen species (ROS and RNS, respectively) (Serrano et al. 2015; Vlot et al. 2009). In parallel with ROS (superoxide anion radical, H_2_O_2_, hydroxyl radical or singlet oxygen) and RNS (mainly nitric oxide (NO) and peroxynitrite) production, SA can also inhibit ROS-scavenging systems, such as the activity of certain antioxidant enzymes. In this way, it contributes to the initiation of programmed cell death (PCD) in the infected tissues (Khan et al. 2014; Poór et al. 2017). Despite the extensive research of cell death mechanisms in plants, the role of SA in ROS and RNS generation during cell death initiation at cell organelle level is not unravelled yet.

Mitochondria have an important role in the initiation of PCD (Lam et al. 2001). By reducing molecular oxygen to superoxide anion radical, complex I and III of the mitochondrial electron transport chain (ETC) are the major sources of ROS production (Blokhina and Fagerstedt 2010; Møller 2001), while complex III and IV are able to reduce nitrite anion to NO under hypoxic conditions. However, there is no convincing evidence for the synthesis of NO by nitric oxide synthase-like (NOS-like) activities in higher plants mitochondria (Gupta and Igamberdiev 2016; Gupta et al. 2011). During HR, the normal function of mitochondria is perturbed. Several harmful changes can be observed in the morphology and functions such as the respiration, ATP synthesis, ROS and NO production or in the membrane potential (ΔΨ_m_) of mitochondria (Nie et al. 2015). The integrity of the mitochondrial inner membrane is crucial for optimal mitochondrial function. In response to cell death stimuli, the outer membrane becomes permeable to water and to large molecules (> 1.5 kDa) after the opening of permeability transition (PT) pores, which leads to the collapse of proton gradient on the inner membrane. In parallel with these changes, the influx of water causes swelling of mitochondria. Moreover, cytochrome *c* (cyt *c*), a component of mitochondrial ETC is also released into the cytosol through the PT pore that contributes to the initiation of PCD (Camacho-Pereira et al. 2009; Godbole et al. 2013; Sarowar et al. 2008; Sun et al. 2008). The composition of the mitochondrial PT pore is under strong debate (Van Aken and Van Breusegem 2015). Based on the model of Kusano et al. (2009) the voltage-dependent anion channel (VDAC), the major protein of the outer mitochondrial membrane and the adenine nucleotide transporter (ANT) in the inner mitochondrial membrane are integral parts of PT pore. Mitochondrial hexokinases (HXKs) can inhibit the PT pore opening by binding to the VDAC at the cytosolic surface and the enzyme protein may act as a plug by blocking the channel (Van Aken and Van Breusegem 2015). SA can affect mitochondrial function in a dose-dependent manner by acting directly on complex III and inhibiting the mitochondrial electron transport and oxidative phosphorylation (Belt et al. 2017; Norman et al. 2004; Shugaev et al. 2014; Xie and Chen 1999). Moreover, SA induced a quick burst of ROS, which can be derived from several cell compartments such as apoplast, chloroplasts or mitochondria and caused the depolarisation of mitochondrial ΔΨ_m_ in the Arabidopsis leaf tissues and protoplasts (Nie et al. 2015). It triggered the release of cyt *c* from the mitochondria to the cytosol in an Arabidopsis cell culture (García-Heredia et al. 2008) and in soybean seedlings (Matos et al. 2009).

Mitochondrial HXKs (mtHXKs) participate in the events that lead to PCD by regulating ROS production and cyt *c* release from mitochondrial intermembrane space (Camacho-Pereira et al. 2009; Kim et al. 2006). Plant HXKs are recognized as multi-function proteins (Aguilera-Alvarado and Sánchez-Nieto 2017). They are the only proteins that are able to phosphorylate glucose (Glc) in plants and they were identified as Glc sensor. Based on their cellular localization (Giegé et al. 2003; Graham et al. 2007; Salvato et al. 2014), four types of HXKs (A–D) were described in plants with different metabolic function. In tomato four *HXK* genes, the mitochondrial *SlHXK1-3* and the plastid-localised *SlHXK4* were identified (Poór et al. 2018). MtHXKs belong to type B hexokinases, which contain a hydrophobic helix at the N terminal attaching the protein to the outside of the mitochondrial outer membrane. Their activity shows strong inhibition in the presence of *N*-acetylglucosamine (NAG), the derivate of Glc or mannoheptulose. Increase in the Glc phosphorylation activity of mtHXKs may reduce cell death by the inhibition of mitochondrial PT pore and by a more efficient Glc metabolism due to better access to ATP (Camacho-Pereira et al. 2009; Godbole et al. 2013; Sarowar et al. 2008; Sun et al. 2008). Control of Glc abundance and signaling by HXK contributes not only to the defence mechanisms but also to the regulation of plant development in accordance with several phytohormones such as: auxin, abscisic acid, gibberellic acid and brassinosteroids (Aguilera-Alvarado and Sánchez-Nieto 2017; Claeyssen and Rivoal 2007; Granot et al. 2013; Moscatello et al. 2017). Based on immunochemical and activity determination, surface plasmon resonance analysis and planar lipid bilayer VDAC-activity analysis, methyl jasmonate (MeJA) can bind to and detach mtHXKs from the mitochondrial membranes (Goldin et al. 2008), which can contribute to ROS accumulation, alterations in mitochondrial movements and morphology, and finally to cell death (Zhang and Xing 2008). In contrast to HXK loss from mitochondrial outer surface, *HXK* overexpression protected tobacco plants against the bacterial pathogen *Pseudomonas syringae* (Sarowar et al. 2008). SA can also regulate the activity and the expression of HXKs (Bruggeman et al. 2015). *HXK1* expression was stimulated after treatment with 5 mM SA in *Nicotiana benthamiana*, while 0.1 mM SA decreased the Glc phosphorylating activity of HXKs and increased the Glc content in tomato leaves after 24 h (Poór et al. 2011).

Although the role of mtHXK activity in the regulation of mitochondrial respiration and ROS production was investigated in plants (Camacho-Pereira et al. 2009), SA-mediated changes in HXK activity and gene expression, as well as HXK-dependent changes in mitochondrial ROS and NO production have not been explored yet in full detail. In this article, comparative biochemical and molecular analyses of HXKs were carried out in time course experiments after sublethal (0.1 mM) and cell death-inducing (1 mM) SA treatments in order to reveal their putative role in the initiation of cell death in tomato leaves. The appearance of cell death symptoms in tissues of tomato plants exposed to these SA concentrations have already been studied in our group (Kovács et al. 2016; Poór et al. 2013). It was found that the first markers of PCD appeared 24 h after SA treatment, so this point seemed to be suitable to compare the differences between the effects of the two SA concentrations. In this paper we focused on the SA-induced physiological changes in the leaves, which can lead to PCD, on the activity and the expression of HXK genes at tissue level, as well as on the changes in mitochondrial morphology in response to SA. However, the main question of this work is whether mtHXKs can control ROS and NO production of leaf mitochondria prepared from SA-treated plants.

## Materials and methods

### Plant material

Germination of tomato plants (*Solanum lycopersicum* L. cv. Ailsa Craig) was started for 3 days at 26 °C under darkness and seedlings were transferred to Perlite for 14 days. Afterwards, plants were grown in a controlled environment under 200 µmol m^−2^ s^−1^ photon flux density (F36W/GRO lamps, OSRAM SYLVANIA, Danvers, MA, USA), with 12/12-h light/dark period, a day/night temperatures of 24/22 °C and a relative humidity of 55–60% for 56 days in hydroponic system (Poór et al. 2011). The experiments were conducted from 9 a.m. (3 h long after light on) and were repeated three times. The samples were prepared from fully expanded leaves in three replicates, 24 h after the 0.1 or 1 mM SA treatments.

### Determination of malondialdehyde (MDA) content and electrolyte leakage (EL) from the leaves

Lipid peroxidation in the leaves was estimated by measuring the thiobarbituric acid-reactive substances (TBARS) with spectrophotometry (Horváth et al. 2015).

Electrolyte leakage (EL) was determined from the leaves as described previously (Sun et al. 2010). Relative EL expresses the actual conductivity (C1) of the incubation medium as a percentage of the total conductivity of boiled tissues (C2) (EL (%) = (C_1_/C_2_) × 100).

### Determination of glucose contents of leaves

0.5 g of leaf tissues was homogenized and boiled for 30 min in ethanol for glucose (Glc) analysis. The homogenate was centrifuged twice at 12,000*g* for 20 min at 4 °C, and the supernatant was assayed for Glc determination by a modular Shimadzu HPLC system (Shimadzu Corp., Kyoto, Japan) equipped with two LC20-AD pumps, a DGU-14A degasser, a SIL-20A autosampler, a CTO-10ASVP column oven and a RID-10A refractive index detector as well as a CBM-20A system controller. For the injection, 15 µL of the samples were applied onto a SphereClone NH_2_ (100 × 4.6 mm 5 µm, Phenomenex, Torrance, CA, USA) column and was separated with isocratic elution using the mixture of HPLC grade water : acetonitrile (30:70, V/V). The flow rate was 1 mL min^−1^ and the oven temperature was 40 °C.

### Morphological changes in mitochondria: transmission electron microscopy (TEM) and post-embedding immunohistochemistry

For post-embedding electron microscopy, leaf segments (5–6 mm) were fixed in 2% paraformaldehyde and 2% glutaraldehyde solution and then further fixed for 1 h in 1% (w/v) OsO_4_. After rinsing in buffer and dehydrating in increasing ethanol concentrations and acetone, they were embedded in Embed812 (Electron Microscopy Sciences, Hatfield, PA, USA). Embedded blocks were used to prepare semithin (0.7 µm) sections, to select the area of interest, and also ultrathin (70 nm) sections, which were mounted on nickel grids. To investigate the effects of SA treatments on the structure of mitochondria in upper palisade mesophyll layer, three grids per block were counterstained with uranyl acetate (Merck, Darmstadt, Germany) and lead citrate (Merck, Darmstadt, Germany) and were examined and photographed with a JEOL JEM 1400 transmission electron microscope (Jeol Ltd., Tokyo, Japan) (Talapka et al. 2016).

Three grids with ultrathin sections from each block were processed for immunogold labelling. Briefly, the grids were incubated overnight with anti-cyt *c* rabbit polyclonal primary antibody (Agrisera, Vännäs, SWEDEN; final dilution 1:1500), followed by protein A-gold-conjugated anti-rabbit (18 nm gold particles, Jackson Immuno Research, West Grove, PA, USA; final dilution 1:20) secondary antibody for 3 h with extensive washing in between steps. The specificity of the immunoreaction was assessed in all cases by omitting the primary antibody from the labelling protocol and incubating the sections only in the protein A-gold conjugated secondary antibody. Sections were counterstained with uranyl acetate (Merck, Darmstadt, Germany) and lead citrate (Merck, Darmstadt, Germany), and then examined and photographed with a JEOL JEM 1400 transmission electron microscope. The number of gold particles assumed to label cyt *c* was determined inside and around mitochondria in the cytosol. Counting was performed on digital photographs at a magnification of 20,000 × with the AnalySIS 3.2 program (Soft Imaging System GmbH, Münster, Germany). Thirty cells per treatments were analysed. Data were expressed as the total number of gold particles per unit area of analysed cell components.

### Isolation of mitochondria and integrity measurements

Isolation of tomato leaf mitochondria was carried out as previously described by Camacho-Pereira et al. (2009). Leaves of tomato plants (100 g) were homogenised in 300 mL of cold extraction buffer containing 10 mM HEPES/Tris buffer (pH 7.4), 0.3 M mannitol, 2 mM EGTA, 5 mM EDTA, 0.3 mM phenylmethylsulfonyl fluoride (PMSF), 20 mM β-mercaptoethanol and 0.1% (w/v) bovine serum albumin (BSA). The homogenate was strained through cheesecloth and centrifuged at 3000*g* for 5 min at 4 °C and after discarding the pellet at 15,000*g* for 15 min at 4 °C. The mitochondrial pellet was resuspended in 5 mL of ice-cold washing buffer (300 mM sucrose, 1 mM EGTA, 0.2 mM PMSF, 10 mM 3-(*N*-morpholino)propanesulfonic acid (MOPS) buffer, pH 7.2), layered on top of tubes. The resuspended mitochondrial fraction was separated in cold washing buffer containing 28% (v/v) Percoll and centrifuged at 40,000*g* for 40 min at 4 °C. After centrifugation, the middle band (fraction 2) containing mitochondria was removed and diluted with 5 mL of ice-cold assay buffer without Percoll (0.3 M mannitol, 1 mM EGTA, 0.2 mM PMSF, 10 mM TRIS, pH 7.2). The dilution and centrifugation were repeated twice at 12,000*g* for 10 min at 4 °C. The final pellet was resuspended in 1 mL of extraction buffer and stored at 4 °C until use. The final protein concentration varied from 10 to 20 mg mL^−1^.

Catalase (CAT) and cytochrome *c* oxidase (COX) was used as a marker to evaluate the purity and integrity of the mitochondrial fractions according to Chen et al. (2009). CAT activity was determined by measuring the exponential decay of 10 mM H_2_O_2_ (Δε_240_ = 39.4 M^−1^ cm^−1^) in 50 mM K-phosphate buffer (pH 7.0) at 240 nm. CAT significantly decreased after the purification, indicating that the mitochondria fraction was free of contamination from peroxisomes. COX was measured in a medium containing 0.3 M sucrose, 5 mM MgCl_2_, 50 µM reduced cyt *c* and 10 mM of K-phosphate buffer (pH 7.0). The reaction was started by the addition of 5 µL of purified mitochondria and the formation of oxidized cyt *c* was detected at 550 nm. 0.04% Triton X-100 was used in order to totally disrupt the mitochondria.

### Detection of reactive oxygen species (ROS) and nitric oxide (NO) in leaf tissues and in isolated mitochondria

ROS and NO production of tomato leaves or that of the mitochondrial fraction was visualized using 10 µM 2,7-dichlorodihydrofluorescein diacetate (H_2_DC-FDA) (Sigma-Aldrich, St. Louis, MO, USA) and 4-amino-5-methylamino-2′,7′-difluorofluorescein diacetate (DAF-FM DA) (Sigma-Aldrich, St. Louis, MO, USA). Leaves were infiltrated with the dyes under vacuum for 30 min in 10 mM Tris–HCl (pH 7.4; 25 °C) in the dark at room temperature and then they were rinsed twice with 10 mM Tris–HCl (pH 7.4; 25 °C). Fluorescence intensity of stained leaf samples was detected with Zeiss Axiowert 200M-type fluorescence microscope (Carl Zeiss Inc., Jena, Germany) equipped with a high-resolution digital camera (Axiocam HR, Carl Zeiss Inc., Jena, Germany). Data were analyzed by AXIOVISION REL. 4.8 software (Carl Zeiss Inc., Munich, Germany) (Poór et al. 2013).

ROS and NO production of mitochondria isolated from leaves of SA-treated plants was determined similarly. Briefly, mitochondria (0.2 mg protein mL^−1^) were incubated in the standard respiration buffer [0.3 M mannitol, 10 mM Tris–HCl, pH 7.2, 3 mM MgSO_4_, 10 mM NaCl, 5 mM KH_2_PO_4_, 0.3 mM β-NAD^+^, 0.1% (w/v) bovine serum albumin (BSA)] supplemented with 10 µM H_2_DC-FDA or DAF-FM DA (Camacho-Pereira et al. 2009) for 10 min. Fluorescence was monitored at excitation and emission wavelengths of 495 nm and 517 nm (H_2_DC-FD) or 500 nm and 515 nm (DAF-FM DA) using a spectrofluorimeter (Hitachi f-4500; Tokyo, Japan). Mitochondrial complex II was activated by 10 mM succinate (Suc) and 0.1 mM adenosine diphosphate (ADP). MtHXK was activated by 5 mM Glc and it was inhibited by 50 µM *N*-acetylglucosamine (NAG).

### RNA extraction, expression analyses by quantitative real-time PCR

Quantitative real-time reverse transcription-PCR (qRT-PCR; Piko Real-Time qPCR System, Thermo Scientific, Waltham, MA, USA) was used to detect the expression pattern of the selected chloroplastic and mitochondrial *HXK* genes (*SlHXK1* (Solyc03g121070): F: 5*′-*TCATCAACCTCCTGGTAAGCA-3′, R: 5*′-*CCTTTTGTCCACCGCATAAT-3′; *SlHXK2* (Solyc06g066440): F: 5′-TCATCCACCTCCTGGTAAGC-3′, R: 5′-TGCCAACCGTGTCATCAAT-3′; *SlHXK3* (Solyc12g008510): R: 5′-TAATGATGGTTCAGGCGTTG-3′, F: 5′-CAGGCACTTTTGGTTGTGTC-3′; *SlHXK4* (Solyc04g081400): F: 5′-GCTGGCAAAAAGGATGTCTAA-3′, R: 5′-CTCCCCATTCGGTATTCACA-3′) mined from Sol Genomics Network (SGN; http://solgenomics.net/) database (Poór et al. 2018). The PCR reaction mixture contained 10 ng cDNA template, 400–400 nM forward and reverse primers, 5 µL of Maxima SYBR Green qPCR Master Mix (2X) (Thermo Scientific, Waltham, MA, USA) and nuclease-free water (denaturation at 95 °C for 7 min, followed by 40 cycles of denaturation at 95 °C for 15 s and annealing extension at 60 °C for 60 s) in 10 µL volume. PikoReal Software 2.2 (Thermo Scientific, Waltham, MA, USA) was used to analyse the data. Tomato 18S rRNA and elongation factor-1α subunit were used as reference genes and the expression data were calculated by the 2^(−∆∆Ct)^ formula.

### Determination of hexokinase (EC 2.7.1.1) activity

Hexokinase activity was measured with glucose substrate according to Whittaker et al. (2001). Soluble proteins were extracted with 1 mL of cold extraction buffer (20 mM KH_2_PO_4_, pH 7.5; 0.5 mM NaEDTA, 5 mM DTT) and after centrifugation (15,000*g*, 15 min, 4 °C) the enzyme activity was measured in the reaction buffer containing 100 mM KH_2_PO_4_ buffer (pH 7.5), 2 mM MgCl_2_, 1 mM NaEDTA, 1 mM ATP, 10 mM glucose, 1 U of glucose-6-phosphate dehydrogenase (EC 1.1.1.49, G6PDH) and 1 U of phosphoglucose isomerase (EC 5.3.1.9, PGI) from baker’s yeast. The measurements were performed by following the increase in absorbance at 340 nm for 5 min at 25 °C (KONTRON, Milano, Italy). Enzyme activities were expressed as nmol min^−1^ mg FM^−1^ and µmol min^−1^ mg protein^−1^ for leaf and mitochondrial samples, respectively. Soluble protein concentration was measured by the method of Bradford (1976).

### Statistical analysis

Experiments were performed in triplicate and repeated three times. The results are expressed as means ± SE. After analyses of variance (ANOVA) multiple comparisons followed by Tukey test were performed with SigmaPlot version 11 software (SYSTAT Software Inc. SPSS). Means were significant if *P* ≤ 0.05.

## Results

### SA-induced physiological changes in the leaves

We aimed to investigate whether the role of HXKs is different in the effects of sublethal (0.1 mM SA) and lethal (1 mM) SA concentrations. In order to reveal these differences, comparative biochemical and molecular analyses of HXKs were carried out in time course experiments using tissue samples or isolated mitochondria prepared from mature leaves of SA treated tomato plants. Firstly, SA-induced physiological changes were determined in the leaves after 24 h. As expected, exogenous SA treatments caused significant accumulation of Glc and both SA treatments caused significantly increased ROS production in the tissues, which were dependent on the applied SA concentration (Table 1). In addition, a significant increase in NO generation was observed after lethal SA treatment in the leaf samples (Table 1). In parallel with the ROS production, both SA treatments enhanced the MDA content deriving from lipid peroxidation. Moreover, the treatments significantly enhanced the electrolyte leakage (EL) from the leaves, which was more than double after the treatment with 1 mM SA compared to the control suggesting the initiation of the cell death program within 24 h in these tissues (Table 1).

**Table 1:** Changes in specific physiological activities in tomato leaves after SA treatments

Treatments	Glucose content (µmol g FM^−1^)	ROS production (% of control)	NO production (% of control)	MDA content (nmol g FM^−1^)	EL (%)
Control	10.3 ± 0.4^b^	100 ± 7.6^c^	100 ± 7.8^b^	27.4 ± 0.5^c^	14.2 ± 0.9^c^
0.1 mM SA	14.4 ± 0.7^a^	122.9 ± 8.5^b^	110.7 ± 13.9^b^	34.3 ± 0.9^b^	24.5 ± 1.3^b^
1 mM SA	14.5 ± 0.5^a^	146.7 ± 18.4^a^	144.1 ± 10.3^a^	54.7 ± 2.6^a^	38.9 ± 2.9^a^

Changes in glucose content, ROS and NO production, MDA content and electrolyte leakage (EL) after 24-h-long 0.1 or 1 mM SA treatments in the leaves tomato. Means ± SE, *n* = 3. Data with different letters indicate significant differences at *P* ≤ 0.05 level (Tukey test)

### SA-induced morphological changes in leaf mitochondria

**Fig. 1:**
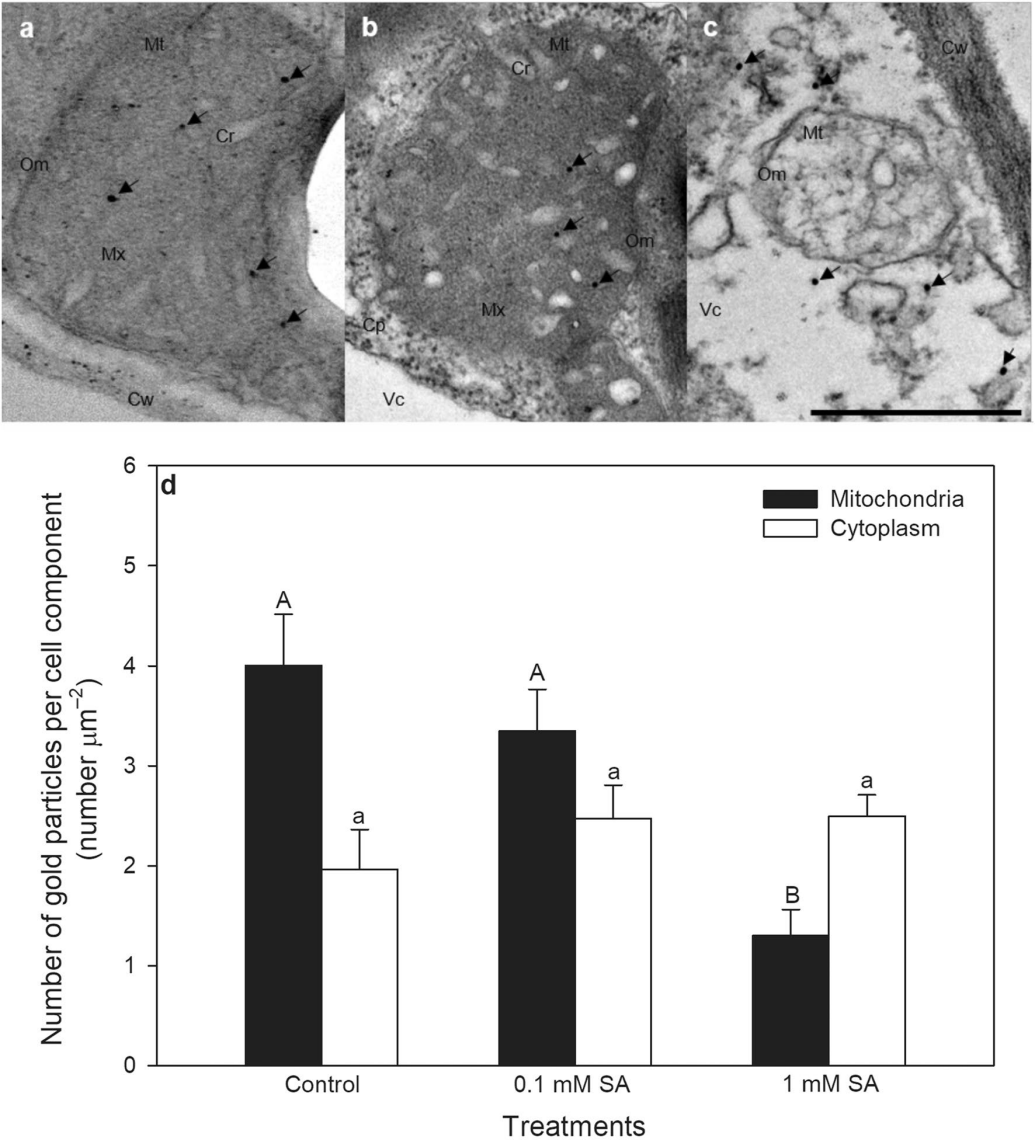
Representative transmission electron micrographs (TEMs) of mitochondria from palisade parenchyma cells of tomato leaves exposed to 0.1 or 1 mM SA for 24 h. **a** Mitochondria with normal organization and structure from a control leaf. **b** Mitochondria in a 0.1 mM SA treated tomato leaves, where SA treatment caused swelling of mitochondrial cristae. **c** Mitochondria in 1 mM SA treated tomato leaves, where SA treatment induced disintegration and vacuolization of mitochondria. **d** The number of gold particles labelling cyt *c* (arrows) in the mitochondria and in the cytoplasm around mitochondria in tomato leaves 24 h after 0.1 or 1 mM SA treatments. *Cp* cytoplasm, *Cr* mitochondrial cristae, *Cw* cell wall, *Mx* mitochondrial matrix, *Mt* mitochondria, *OM* mitochondrial outer membrane, *Vc* vacuole. Means ± SE, *n* = 30. Data with different letters indicate significant differences at *P* ≤ 0.05 level (Tukey test). Bar = 500 nm

To detect the morphological effects of SA on leaf mitochondria and early cytological alterations that induce the execution of cell death, transmission electron micrographs (TEMs) and post-embedding immunohistochemistry were used. TEMs of leaf sections revealed significant changes in mitochondrial structure after 24 h (Fig. 1a–c). SA treatments caused swelling and disorganisation of mitochondrial cristae as well as disintegration and vacuolization of mitochondria, which proved to be more serious at higher SA concentration (Fig. 1c). Because mitochondria are important integrators during the induction of cell death, cyt *c* release from mitochondrial intermembrane space was monitored by post-embedding immunohistochemistry after SA treatments. Mitochondrial cyt *c* levels decreased significantly only in 1 mM SA-treated leaves after 24 h suggesting that the initiation of PCD was started (Fig. 1d). Contrary to the mitochondrial fraction, the cytosolic cyt *c* level around the mitochondria increased only slightly, which was not significant in SA treated plants after 24-h-long hormone exposure (Fig. 1d).

### Effects of SA on tomato HXKs

**Fig. 2:**
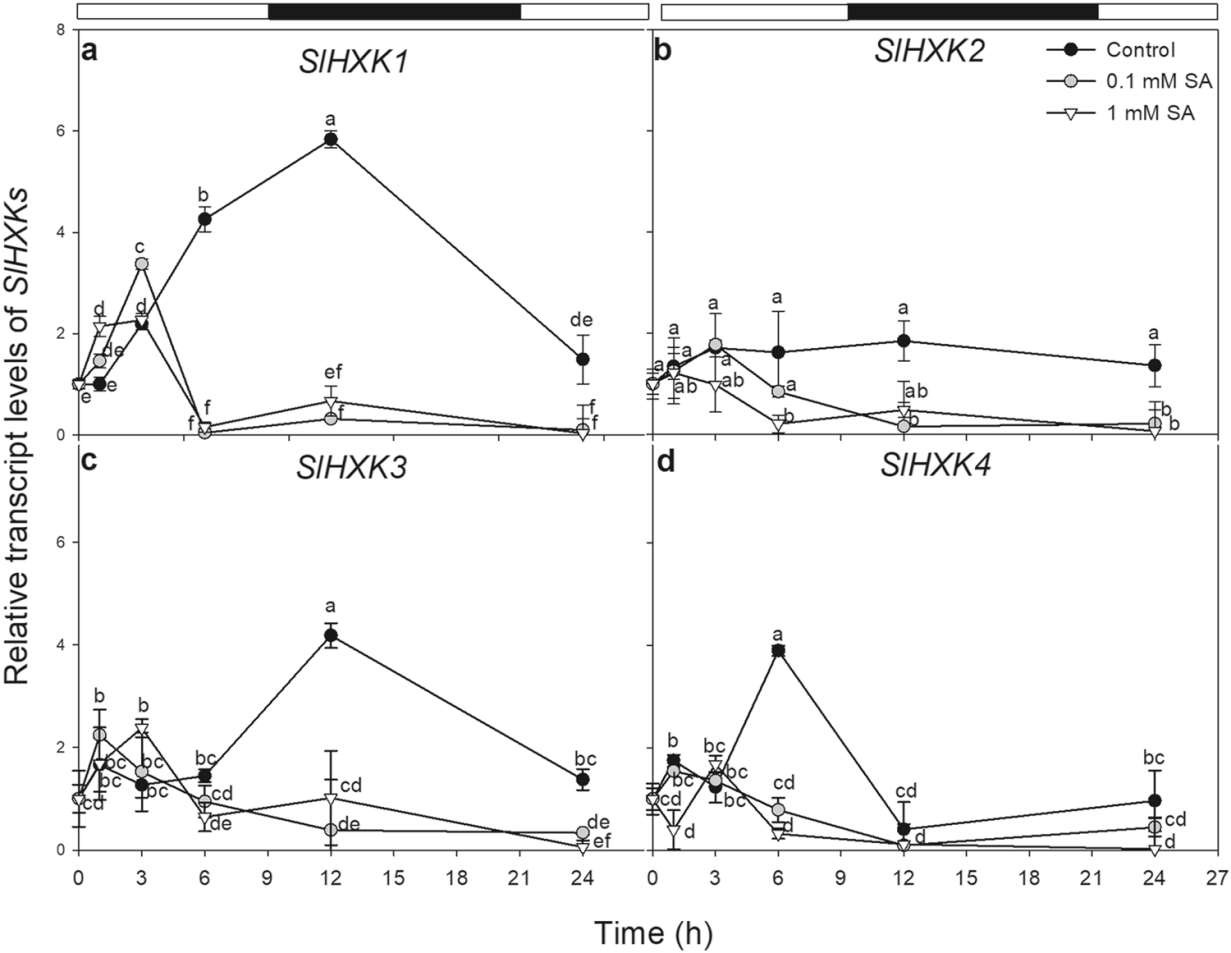
Changes in the relative transcript levels of *SlHXK1* (**a**), *SlHXK2* (**b**), *SlHXK3* (**c**) and *SlHXK4* (**d**) as a function of time after 0.1 or 1 mM SA treatments in the leaves tomato (black circle: control; white circle: 0.1 mM SA; white triangle: 1 mM SA). Means ± SE, *n* = 3. Data with different letters indicate significant differences at *P* ≤ 0.05 level (Tukey test) at the given time point

Since SA was expected to control HXK function, changes in relative transcript amount and activity of tomato HXKs, which catalyse the first step of glucose catabolism in glycolysis, were also investigated in time course experiments. The expression of four tomato *HXK* genes, the mitochondrial *SlHXK1-3* and the plastid-localised *SlHXK4* were examined as a function of time after SA treatments. Under control conditions, transcript levels of HXK-coding genes, especially those of *SlHXK1, 3* and *4* exhibited diurnal fluctuations in the mature leaves of tomato. The expression of *SlHXK4* reached a maximum in the light period and decreased in the dark (Fig. 2d), while the expression of *SlHXK1* and *SlHXK3* was highest in the early dark period under the control condition then decreased until the beginning of the light period (Fig. 2a, c). Both SA treatments significantly inhibited the expression of all *SlHXKs* after 6 h (Fig. 2).

**Fig. 3:**
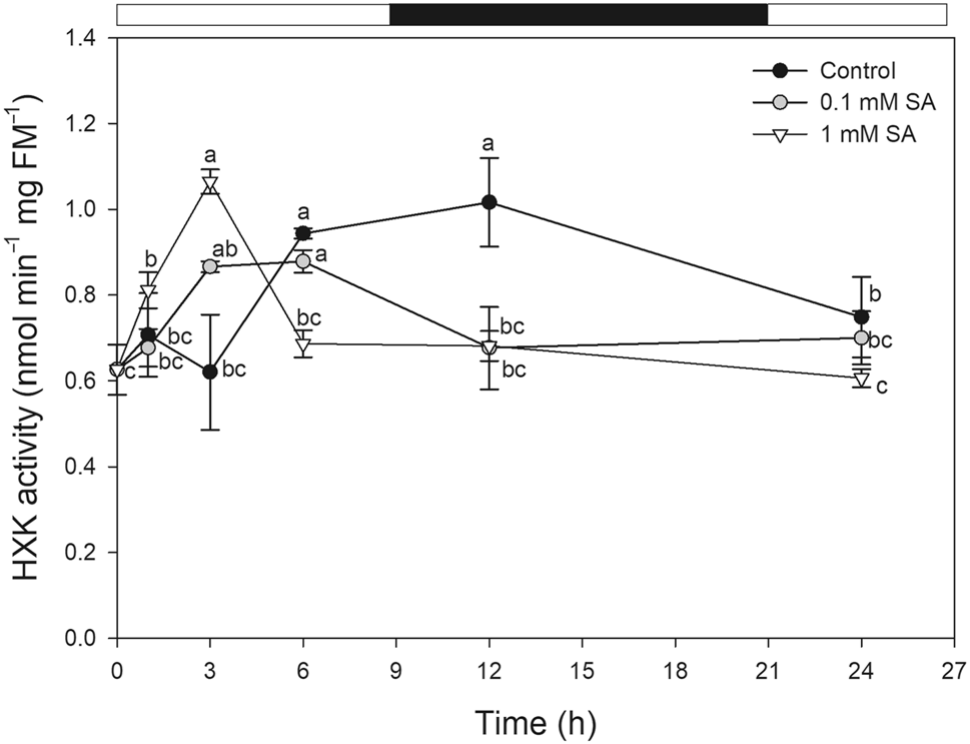
Changes in the total activity of hexokinases (HXK) in the presence of glucose substrate as a function of time after 0.1 or 1 mM SA treatments in leaf extract of tomato plants. Means ± SE, *n* = 3. Data with different letters indicate significant differences at *P* ≤ 0.05 level (Tukey test) at the given time points

In order to reveal whether the decrease in *SlHXK* gene expression in plants treated with SA can lead to changes in Glc metabolism and cell death induction, the total enzymatic activity of HXKs was also determined. The activity of HXKs with Glc substrate showed similar changes as gene expression data. HXK activity increased in the leaves after 3 h then significantly decreased from 6 h after 1 mM SA treatment (Fig. 3). The total HXK activity was also significantly reduced by 0.1 mM SA from 6 h but after 24 h the activity returned to the control level (Fig. 3).

### HXK-dependent mitochondrial ROS and NO production

**Fig. 4:**
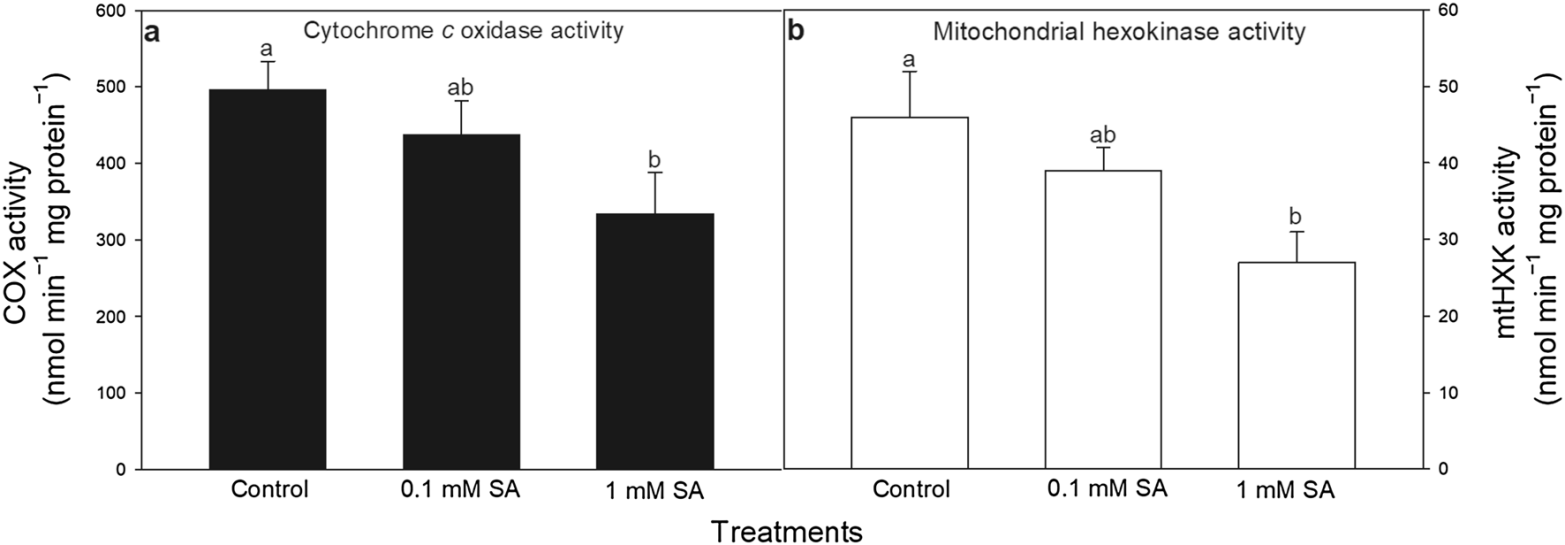
Changes in the activity of cytochrome *c* oxidase (COX; **a**) and mitochondrial hexokinases (mtHXK) in the presence of glucose substrate (**b**) in the mitochondrial fraction of leaves of tomato after 24-h-long 0.1 or 1 mM SA treatments. Means ± SE, *n* = 3. Data with different letters indicate significant differences at *P* ≤ 0.05 level (Tukey test)

The compiled transcriptional and biochemical data suggest that changes in HXK regulation after SA treatments affect the physiological response of leaves including mitochondrial metabolism and function preceding the cell death incidence. Therefore, experiments with isolated mitochondria from leaves of SA treated plants were performed to show that SA-treatments trigger mitochondrial ROS and NO production, which can be dependent on mtHXK activity. Firstly, the activity of COX, the marker enzyme of mitochondria was analysed (Fig. 4a). The decrease in COX activity upon 1 mM SA indicated that high concentration of SA induced mitochondrial disorganisation and initiated cell death in leaves of tomato plants by a pronounced cyt *c* release from mitochondrial intermembrane space to the cytosol (Fig. 1). Moreover, the total mtHXK activity was also measured in isolated mitochondria prepared from plants exposed to 24-h-long SA treatments. MtHXK activity declined to a moderate extent in mitochondria upon 0.1 mM SA and decreased significantly upon 1 mM SA treatment (Fig. 4b).

**Fig. 5:**
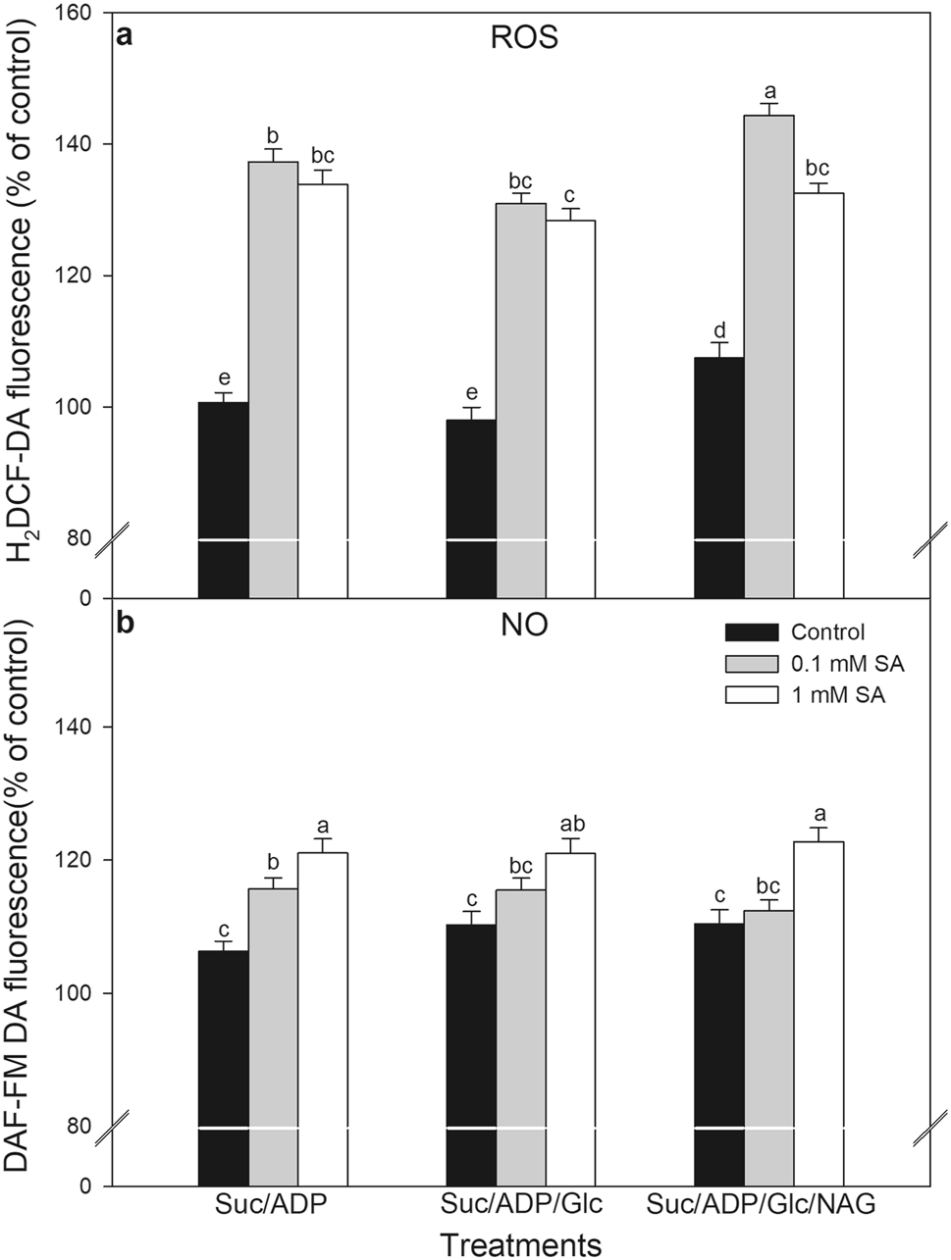
Changes in ROS (**a**) and NO (**b**) production in the presence of different modulators in mitochondrial fraction of leaves of tomato after 24 h 0.1 or 1 mM SA treatments. *Suc* succinate, *ADP* adenosine diphosphate, *Glc* glucose, *NAG N*-acetylglucosamine. Means ± SE in % of control, *n* = 3. Data with different letters indicate significant differences at *P* ≤ 0.05 level (Tukey test)

In the next set of experiments, the role of mtHXK activity in mitochondrial ROS and NO production was evaluated with isolated mitochondria from leaves of 24-h-long-SA treated plants. Firstly, succinate and ADP were added to the mitochondria isolated from leaves of control and SA treated plants to enhance the mitochondrial ETC activity. Succinate accelerated the electron flux into the mitochondrial respiratory electron transport chain at complex II and together with ADP increased the rate of O_2_ consumption, the activity of mitochondrial ETC, the generation of ROS and mtHXK activity (Camacho-Pereira et al. 2009). In our experiments mitochondrial ROS and NO production was significantly induced by both SA treatments in the isolated samples (Fig. 5) and in parallel mtHXK activity was lower than in the control samples (Fig. 4b). The addition of Glc, which is the substrate of HXKs, slightly but not significantly decreased mitochondrial ROS accumulation in samples isolated from SA-treated plants (Fig. 5a). This was reversed by HXK inhibitor N-acetylglucosamine (NAG), that promoted ROS production in control and SA-treated mitochondria especially at lower SA concentration (Fig. 5a). However, these latter treatments did not influence NO emission in the samples, which was significantly increased with increasing SA concentration independently of mtHXK activity (Fig. 5b). These experiments showed that decreased mtHXK activity in the presence of NAG could contribute to the mitochondrial ROS production but not to NO generation, in leaf mitochondria of SA-treated plants. Moreover, 1 mM SA was able to decrease mtHXK activity very significantly, which correlated well with the decline in COX activity. This suggests that the Glc phosphorylating activity of mtHXKs can control ROS production in the case of intact mitochondria (0.1 mM SA), but this effect disappeared in highly damaged cell compartments after 1 mM SA treatment.

## Discussion

Our earlier studies revealed that 1 mM SA initiated the cell death program within 24 h, which was accompanied by lesion formation in the leaf mesophyll cells of tomato plants. It correlated with a decline photosynthetic activity and CO_2_ assimilation in SA-treated plants (Poór et al. 2011). In the present study, the role of HXKs was investigated in SA-induced physiological responses and cell death process in the first 24 h after SA application. Based on the results, the sublethal and lethal concentrations of SA elevated endogenous Glc content in the leaves. Glc as a substrate can influence the glucokinase activity of HXKs thus it has an effect on mitochondrial and photosynthetic sugar metabolism (Zhang and Xing 2008; Bolouri-Moghaddam et al. 2010).

High ROS and NO levels that follow SA accumulation are among the initial signs of HR-like cell death (Serrano et al. 2015; Vlot et al. 2009). However, both applied SA concentrations generated high ROS levels and 1 mM SA induced high NO production in leaves 24 h after SA treatment. It has been obtained by several authors that the dysfunction of mitochondrial and photosynthetic ETC results in excess ROS and NO production (Blokhina and Fagerstedt 2010; Gupta et al. 2011; Møller 2001). SA can decrease the maximum and effective quantum yields of PSII photochemistry and can inhibit the linear electron transport rate in the chloroplasts (Janda et al. 2014; Nazar et al. 2011; Poór et al. 2011). At high concentration it can act directly on the complex III in mitochondrial inner membrane and can also inhibit the mitochondrial electron transport (Norman et al. 2004; Xie and Chen 1999). This may lead to the formation of ROS and NO in both cell organelles. In intact leaves, however, ROS can be produced by a number of enzymes, such as NADPH oxidase, polyamine oxidases and peroxidases in various cell compartments, which can also be controlled by SA (Poór et al. 2017; Takács et al. 2018). SA also inhibits ROS-scavenging systems such as the activity of certain antioxidant enzymes (e.g. that of catalase), which results in the further enhancement in ROS accumulation (Khan et al. 2014; Tari et al. 2015). Beside chloroplasts and mitochondria, the cytoplasm, peroxisomes and the apoplast may be the sites of NO production, too, which can also be controlled by SA (Takács et al. 2016). Oxidative degradation of lipids caused by SA destroys cell membranes, resulting in cell damage and loss of cell integrity (Poór et al. 2017). Both applied SA treatments increased lipid peroxidation based on MDA content and electrolyte leakage (EL) from leaf tissues in our system, but it was much serious and promoted cell death only at 1 mM SA. Similar results were reported by Kovács et al. (2016), who detected along with the increase in EL the induction of the expression of various cysteine proteases (e.g. vacuolar processing enzyme) and enhanced proteolysis in this system 24 h after SA treatment.

Cell death in plant leaves is preceded by various early cytological features: specific morphological changes, chromatin aggregation and DNA fragmentation. Changes in morphology and thus activity and metabolism of mitochondria is also part of this process (Lam et al. 2001) and loss of mitochondrial integrity and cyt *c* release from mitochondria take place before cell death execution (Dat et al. 2003). In this article we demonstrated first, that SA exposure caused a concentration-dependent disorganisation of mitochondrial cristae, swelling and vacuolization of mitochondria, as well as cyt *c* release from the mitochondrial intermembrane space in palisade parenchyma cells of tomato leaves 24 h after 1 mM SA treatment.

MtHXKs attached to the outer mitochondrial membrane can prevent cyt *c* release from the intermembrane space of mitochondria by inhibiting the PT pore opening and thereby they control the cell death mechanism (Camacho-Pereira et al. 2009; Godbole et al. 2013; Sarowar et al. 2008; Sun et al. 2008). However, it is not known whether mtHXK activity influences ROS and NO production and as a consequence cyt *c* release from isolated mitochondria prepared from SA-treated plants.

In tomato, four *HXK* genes (*SlHXK1-4*) were identified (Damari-Weissler et al. 2006) and according to our results all of them, comprising mtHXKs, were downregulated after SA treatments in the leaves. In addition, the total HXK activity with Glc substrate decreased after the exogenous SA application. This decline in the transcription and activity of HXKs is in good agreement with the measured high Glc levels in the leaf tissues. Moreover, inhibition of the HXK activity or low levels of mtHXKs could contribute to the SA-induced mitochondrial ROS production and cyt *c* release in the leaf cells.

In accordance with the results obtained in leaf tissues, mtHXK activity significantly decreased in the mitochondria isolated from the leaves exposed to SA. Thus, inhibition of mtHXK activity or low levels of mtHXK expression can contribute to higher mitochondrial ROS in the 24-h samples. In addition, decrease in COX activity upon 1 mM SA indicated the disintegration of mitochondrial envelop membranes within 24 h.

The mitochondrial ETC, the major site of ROS production and ATP synthesis are highly dependent on the ΔΨ_m_ generated by the proton gradient across the inner mitochondrial membrane (Møller 2001). ΔΨ_m_ dissipation was observed in sugar beet (*Beta vulgaris* L.) and etiolated seedling cotyledons of yellow lupine (*Lupinus luteus* L.) after SA exposure, because SA can uncouple and inhibit the respiratory electron transport chain depending on the applied concentration and duration of the treatment (Shugaev et al. 2014). Camacho-Pereira et al. (2009) demonstrated using potato tuber tissues (*Solanum tuberosum* L.) that mtHXK activity plays a key “preventive antioxidant” role by reducing mitochondrial ROS generation through a steady-state ADP recycling mechanism. In parallel, the increase in O_2_ consumption after mtHXK activation by Glc was observed suggesting that the ETC was also activated in the presence of Glc (Camacho-Pereira et al. 2009). Mitochondrial ROS and NO production was also elevated in our system after addition of Suc and ADP to the isolated mitochondria. Nevertheless, the mitochondrial ROS production was reduced only slightly by the addition of Glc in these SA-treated samples. However, the application of NAG, the inhibitor of HXK significantly enhanced ROS production of control and SA treated mitochondria especially at 0.1 mM SA. Although NO production displayed significant SA concentration dependency, the application of Glc or NAG did not affect NO emission from these samples. Moreover, the source and the mechanism of NO production is not known. Mitochondria from leaf tissues of several species (e.g. from pea) did not generate significant quantities of NO even under anoxia but in other species such as tobacco and barley, the emission of NO in the gas phase of the suspension was found in nanomolar range (per mg protein and per hour) measured by ozone collision gas phase chemiluminescence (Gupta et al. 2011). Since constitutive NOS-like activity cannot be found in plant mitochondria, the L-Arginine derived NO formation by NOS and the nitrite reduction by mitochondrial ETC in the absence of added nitrite can be excluded in our system. Thus the chemical nature of DAF-FM fluorescence is not known in this system.

These experiments demonstrated for the first time that decreased mtHXK activity could contribute to the mitochondrial ROS production but not to NO generation in SA-treated leaves. This observation suggests that SA can contribute to cell death induction in tomato leaves by modulating mtHXK transcription and activity, which can influence mitochondrial functions resulting in ROS production, while NO accumulation seems to be independent of mtHXK activity but it is strongly regulated by SA.

## References

[CR1] Aguilera-Alvarado GP, Sánchez-Nieto S (2017). Plant hexokinases are multifaceted proteins. Plant Cell Physiol.

[CR2] Belt K, Huang S, Thatcher LF, Casarotto H, Singh K, Van Aken O, Millar AH (2017). Salicylic acid-dependent plant stress signalling via mitochondrial succinate dehydrogenase. Plant Physiol.

[CR3] Blokhina O, Fagerstedt KV (2010). Reactive oxygen species and nitric oxide in plant mitochondria: origin and redundant regulatory systems. Physiol Plant.

[CR4] Bolouri-Moghaddam MR, Le Roy K, Xiang L, Rolland F, Van den Ende W (2010). Sugar signalling and antioxidant network connections in plant cells. FEBS J.

[CR5] Bradford MM (1976). A rapid and sensitive method for the quantitation of microgram quantities of protein utilizing the principle of protein-dye binding. Anal Biochem.

[CR6] Bruggeman Q, Prunier F, Mazubert C, de Bont L, Garmier M, Lugan R, Delarue M (2015). Involvement of *Arabidopsis* hexokinase1 in cell death mediated by myo-inositol accumulation. Plant Cell.

[CR7] Camacho-Pereira J, Meyer LE, Machado LB, Oliveira MF, Galina A (2009). Reactive oxygen species production by potato tuber mitochondria is modulated by mitochondrially bound hexokinase activity. Plant Phys.

[CR8] Chen X, Wang Y, Li J, Jiang A, Cheng Y, Zhang W (2009). Mitochondrial proteome during salt stress-induced programmed cell death in rice. Plant Physiol Biochem.

[CR9] Claeyssen É, Rivoal J (2007). Isozymes of plant hexokinase: occurrence, properties and functions. Phytochemistry.

[CR10] Damari-Weissler H, Kandel-Kfir M, Gidoni D, Mett A, Belausov E, Granot D (2006). Evidence for intracellular spatial separation of hexokinases and fructokinases in tomato plants. Planta.

[CR11] Dat JF, Pellinen R, Van De Cotte B, Langebartels C, Kangasjärvi J, Inzé D, Van Breusegem F (2003). Changes in hydrogen peroxide homeostasis trigger an active cell death process in tobacco. Plant J.

[CR12] García-Heredia JM, Hervás M, Miguel A, Navarro JA (2008). Acetylsalicylic acid induces programmed cell death in *Arabidopsi*s cell cultures. Planta.

[CR13] Giegé P, Heazlewood JL, Roessner-Tunali U, Millar AH, Fernie AR, Leaver CJ, Sweetlove LJ (2003). Enzymes of glycolysis are functionally associated with the mitochondrion in Arabidopsis cells. Plant Cell.

[CR14] Godbole A, Dubey AK, Reddy PS, Udayakumar M, Mathew MK (2013). Mitochondrial VDAC and hexokinase together modulate plant programmed cell death. Protoplasma.

[CR15] Goldin N, Arzoine L, Heyfets A, Israelson A, Zaslavsky Z, Bravman T, Flescher E (2008). Methyl jasmonate binds to and detaches mitochondria-bound hexokinase. Oncogene.

[CR16] Graham JW, Williams TC, Morgan M, Fernie AR, Ratcliffe RG, Sweetlove LJ (2007). Glycolytic enzymes associate dynamically with mitochondria in response to respiratory demand and support substrate channeling. Plant Cell.

[CR17] Granot D, David-Schwartz R, Kelly G (2013). Hexose kinases and their role in sugar-sensing and plant development. Front Plant Sci.

[CR18] Gupta KJ, Igamberdiev AU (2016). Reactive nitrogen species in mitochondria and their implications in plant energy status and hypoxic stress tolerance. Front Plant Sci.

[CR19] Gupta KJ, Igamberdiev AU, Manjunatha G, Segu S, Moran JF, Neelawarne B, Kaiser WM (2011). The emerging roles of nitric oxide (NO) in plant mitochondria. Plant Sci.

[CR20] Hayat Q, Hayat S, Irfan M, Ahmad A (2010). Effect of exogenous salicylic acid under changing environment: a review. Environ Exp Bot.

[CR21] Horváth E, Szalai G, Janda T (2007). Induction of abiotic stress tolerance by salicylic acid signaling. J Plant Growth Regul.

[CR22] Horváth E, Csiszár J, Gallé Á, Poór P, Szepesi Á, Tari I (2015). Hardening with salicylic acid induces concentration-dependent changes in abscisic acid biosynthesis of tomato under salt stress. J Plant Physiol.

[CR23] Janda T, Gondor OK, Yordanova R, Szalai G, Pál M (2014). Salicylic acid and photosynthesis: signalling and effects. Acta Physiol Plant.

[CR01] Khan MIR, Khan NA (2013). Salicylic acid and jasmonates: approaches in abiotic stress tolerance. J Plant Biochem Physiol.

[CR24] Khan MIR, Asgher M, Khan NA (2014). Alleviation of salt-induced photosynthesis and growth inhibition by salicylic acid involves glycinebetaine and ethylene in mungbean (*Vigna radiata* L). Plant Physiol Biochem.

[CR25] Khan MIR, Fatma M, Per TS, Anjum NA, Khan NA (2015). Salicylic acid-induced abiotic stress tolerance and underlying mechanisms in plants. Front Plant Sci.

[CR26] Kim M, Lim JH, Ahn CS, Park K, Kim GT, Kim WT, Pai HS (2006). Mitochondria-associated hexokinases play a role in the control of programmed cell death in *Nicotiana benthamiana*. Plant Cell.

[CR27] Kovács J, Poór P, Szepesi Á, Tari I (2016). Salicylic acid induced cysteine protease activity during programmed cell death in tomato plants. Acta Biol Hung.

[CR28] Kusano T, Tateda C, Berberich T, Takahashi Y (2009). Voltage-dependent anion channels: their roles in plant defense and cell death. Plant Cell Rep.

[CR29] Lam E, Kato N, Lawton M (2001). Programmed cell death, mitochondria and the plant hypersensitive response. Nature.

[CR30] Matos AR, Mendes AT, Scotti-Campos P, Arrabaça JD (2009). Study of the effects of salicylic acid on soybean mitochondrial lipids and respiratory properties using the alternative oxidase as a stress-reporter protein. Physiol Plant.

[CR31] Møller IM (2001). Plant mitochondria and oxidative stress: electron transport, NADPH turnover, and metabolism of reactive oxygen species. Annu Rev Plant Biol.

[CR32] Moscatello S, Proietti S, Buonaurio R, Famiani F, Raggi V, Walker RP, Battistelli A (2017). Peach leaf curl disease shifts sugar metabolism in severely infected leaves from source to sink. Plant Physiol Biochem.

[CR33] Nazar R, Iqbal N, Syeed S, Khan NA (2011). Salicylic acid alleviates decreases in photosynthesis under salt stress by enhancing nitrogen and sulfur assimilation and antioxidant metabolism differentially in two mungbean cultivars. J Plant Physiol.

[CR34] Nie S, Yue H, Zhou J, Xing D (2015). Mitochondrial-derived reactive oxygen species play a vital role in the salicylic acid signaling pathway in *Arabidopsis thaliana*. PLoS One.

[CR35] Norman C, Howell KA, Millar AH, Whelan JM, Day DA (2004). Salicylic acid is an uncoupler and inhibitor of mitochondrial electron transport. Plant Physiol.

[CR36] Poór P, Gémes K, Horváth F, Szepesi A, Simon ML, Tari I (2011). Salicylic acid treatment via the rooting medium interferes with stomatal response, CO_2_ fixation rate and carbohydrate metabolism in tomato, and decreases harmful effects of subsequent salt stress. Plant Biol.

[CR37] Poór P, Kovács J, Szopkó D, Tari I (2013). Ethylene signaling in salt stress-and salicylic acid-induced programmed cell death in tomato suspension cells. Protoplasma.

[CR38] Poór P, Takács Z, Bela K, Czékus Z, Szalai G, Tari I (2017). Prolonged dark period modulates the oxidative burst and enzymatic antioxidant systems in the leaves of salicylic acid-treated tomato. J Plant Physiol.

[CR39] Poór P, Takács Z, Patyi G, Borbély P, Bencsik O, Szekeres A, Tari I (2018). Dark-induced changes in the activity and the expression of tomato hexokinase genes depend on the leaf age. S Afr J Bot.

[CR40] Rivas-San Vicente M, Plasencia J (2011). Salicylic acid beyond defence: its role in plant growth and development. J Exp Bot.

[CR41] Salvato F, Havelund JF, Chen M, Rao RSP, Rogowska-Wrzesinska A, Jensen ON, Gang DR, Thelen JJ, Møller IM (2014). The potato tuber mitochondrial proteome. Plant Physiol.

[CR42] Sarowar S, Lee JY, Ahn ER, Pai HS (2008). A role of hexokinases in plant resistance to oxidative stress and pathogen infection. J Plant Biol.

[CR43] Serrano I, Romero-Puertas MC, Sandalio LM, Olmedilla A (2015). The role of reactive oxygen species and nitric oxide in programmed cell death associated with self-incompatibility. J Exp Bot.

[CR44] Shugaev AG, Butsanets PA, Andreev IM, Shugaeva NA (2014). Effect of salicylic acid on the metabolic activity of plant mitochondria. Russ J Plant Physiol.

[CR45] Sun L, Shukair S, Naik TJ, Moazed F, Ardehali H (2008). Glucose phosphorylation and mitochondrial binding are required for the protective effects of hexokinases I and II. Mol Cell Biol.

[CR46] Sun J, Li L, Liu M, Wang M, Ding M, Deng S, Lu C, Zhou X, Shen X, Zheng X, Chen S (2010). Hydrogen peroxide and nitric oxide mediate K^+^/Na^+^ homeostasis and antioxidant defense in NaCl stressed callus cells of two contrasting poplars. Plant Cell Tissue Organ Cult.

[CR47] Takács Z, Poór P, Tari (2016). Comparison of polyamine metabolism in tomato plants exposed to different concentrations of salicylic acid under light or dark conditions. Plant Physiol Biochem.

[CR48] Takács Z, Poór P, Borbély P, Czékus Z, Szalai G, Tari I (2018). H_**2**_O_**2**_ homeostasis in wild-type and ethylene-insensitive *Never ripe* tomato in response to salicylic acid treatment in normal photoperiod and in prolonged darkness. Plant Physiol Biochem.

[CR49] Talapka P, Berkó A, Nagy LI, Chandrakumar L, Bagyánszki M, Puskás LG, Bódi N (2016). Structural and molecular features of intestinal strictures in rats with Crohn’s-like disease. World J Gastroenterol.

[CR50] Tari I, Csiszár J, Horváth E, Poór P, Takács Z, Szepesi Á (2015). The alleviation of the adverse effects of salt stress in the tomato plant by salicylic acid shows a time-and organ-specific antioxidant response. Acta Biol Cracov Bot.

[CR51] Van Aken O, Van Breusegem F (2015). Licensed to kill: mitochondria, chloroplasts, and cell death. Trends Plant Sci.

[CR52] Vlot AC, Dempsey DMA, Klessig DF (2009). Salicylic acid, a multifaceted hormone to combat disease. Annu Rev Phytopathol.

[CR02] Whittaker A, Bochicchio A, Vazzana C, Lindsey G, Farrant J (2001). Changes in leaf hexokinase activity and metabolite levels in response to drying in the desiccation‐tolerant species Sporobolus stapfianus and Xerophyta viscosa. J Exp Bot.

[CR53] Xie Z, Chen Z (1999). Salicylic acid induces rapid inhibition of mitochondrial electron transport and oxidative phosphorylation in tobacco cells. Plant Physiol.

[CR54] Yan S, Dong X (2014). Perception of the plant immune signal salicylic acid. Curr Opin Plant Biol.

[CR55] Zhang L, Xing D (2008). Methyl jasmonate induces production of reactive oxygen species and alterations in mitochondrial dynamics that precede photosynthetic dysfunction and subsequent cell death. Plant Cell Physiol.

